# Family E-Chat Group Use Was Associated with Family Wellbeing and Personal Happiness in Hong Kong Adults amidst the COVID-19 Pandemic

**DOI:** 10.3390/ijerph18179139

**Published:** 2021-08-30

**Authors:** Wei-Jie Gong, Bonny Yee-Man Wong, Sai-Yin Ho, Agnes Yuen-Kwan Lai, Sheng-Zhi Zhao, Man-Ping Wang, Tai-Hing Lam

**Affiliations:** 1 School of Public Health, The University of Hong Kong, Hong Kong, China; gweijie@connect.hku.hk (W.-J.G.); wongbonny@hotmail.com (B.Y.-M.W.); syho@hku.hk (S.-Y.H.); hrmrlth@hku.hk (T.-H.L.); 2 School of Nursing, The University of Hong Kong, Hong Kong, China; agneslai@hku.hk (A.Y.-K.L.); lubabezz@connect.hku.hk (S.-Z.Z.)

**Keywords:** information and communication technology, instant messaging, family e-chat group, family communication, family wellbeing, personal happiness

## Abstract

Instant messaging (IM) is increasingly used for family communication amidst the COVID-19 pandemic. However, evidence remains scarce on how family e-chat groups were used and their associations with family and individual wellbeing amidst the pandemic. The numbers of family e-chat groups, functions used, and messages sent and received daily in groups were reported by 4890 adults in May 2020, and their associations with family wellbeing and personal happiness and the mediation effect of family communication quality were examined. Results showed that sending/receiving text messages was most commonly used, followed by receiving/sending photos/pictures, making voice calls, receiving/sending short videos and voice messages, and making video calls. Women and older people used more non-text functions. Higher levels of family wellbeing and personal happiness were associated with having more groups, receiving/sending photos/pictures, video calls, more IM functions used, and more IM messages received/sent daily. Forty-six point two to seventy-five point five percent of their associations with more groups and more functions used were mediated by family communication quality. People having more family e-chat groups and using more IM functions may be more resilient amidst the pandemic, while those without or with low use of family e-chat groups amidst the pandemic would need more attention and assistance in the presence of social distancing.

## 1. Introduction

Family is rated the most important among six aspects of life, surpassing friends, leisure time, politics, work, and religion [1]. Family wellbeing, usually conceptualized as family functioning, family life satisfaction, or family quality of life [2,3], not only enhances the physical and mental health of individuals across the lifespan but also fosters stable and cohesive societies [4]. Family wellbeing is affected by external and internal factors irrespective of the cultural context. External factors include the availability of social and community resources, such as income, social networks, and medical services, which build the material and social foundation of family life [5]. Internally, family communication is central to sustaining family relationships and family wellbeing regardless of family structure [6]. Quality family communication through verbal and nonverbal interactions enables members to share attitudes and beliefs, be related, act with cohesion and flexibility, achieve satisfaction, and share information inside and outside the family boundaries [6,7].

Internet use and information and communication technology (ICT) have dramatically changed interpersonal communication within the family [8,9]. Advanced ICT applications, such as WhatsApp or WeChat, provide convenient instant messaging (IM) functions that allow family members to connect and share information in real-time [10,11,12]. Specifically, e-chat groups in these applications allow three or more users to simultaneously share texts, images, voice messages, short videos, and even make video calls at low or no costs. Previous studies found more family communication using IM messages and video calls was associated with higher levels of family wellbeing [13,14].

The coronavirus disease 2019 (COVID-19) pandemic disrupts the external factors of family wellbeing, posing grave threats to both individuals and families by the interruption of daily routines, financial insecurity, lockdown, physical distancing, and social disruptions [15,16]. Increased family-related mental burdens have been reported. Over 75.0% of Chinese reported concerns about family members’ health during the initial outbreak in China [17]. A subsequent report in Canada also showed that 32.0% of respondents were very or extremely concerned about family stress from confinement [18]. In Hong Kong, 33.3% of respondents reported increased family negative emotion, 18.9% reported decreased family happiness [19], and the prevalence of individual unhappiness doubled that in 2016 and 2017 [20]. While face-to-face communication has reduced with physical distancing restrictions, digital communication via IM tools has increased [21,22].

The present study was informed by two related theoretical frameworks. First, Prime and Wade’s framework emphasizes the importance of family communication amidst the COVID-19 pandemic on the basis of Walsh’s family resilience framework [16,23]. Constructive and effective family communication preserves and nourishes relationships and shares beliefs to cope with risks during social disruption [16]. Second, Castellacci and Tveito’s theoretical framework on Internet use and wellbeing posits that Internet use shapes wellbeing through creating new activities and improved forms of remote communication [24]. Family e-chat groups using various IM functions help maintain instant interactions and avoid mental isolation in periods of physical distancing. We hypothesized that IM use in the family may be associated with family and individual wellbeing through communication quality.

In Hong Kong, 98.4% of Internet users took online social activities as a major purpose for getting online in 2018 [25], and the smartphone penetration rate increased to 91.5% in 2019 [26]. We searched PubMed and Web of Science using keywords of “2019 nCoV”, “COVID-19”, “SARS-CoV-2”, “instant messages”, “e-chat group”, “family”, and “happiness” up to 16 August 2021 and found no survey reports on how people make use of family e-chat groups amidst the COVID-19 pandemic. Only one study reported that, in 2017, 72.0% of Hong Kong adults had at least one family e-chat group, and 72.0% and 83.7% of them received and sent at least one message daily [14]. No study has reported the IM functions used or contents delivered in family e-chat groups before and amidst the pandemic. The aims of the present study were to examine the use of family e-chat groups, especially different IM functions, amidst the COVID-19 pandemic and the associations with family wellbeing and personal happiness, and the mediating effects of family communication on these associations.

## 2. Materials and Methods

### 2.1. Study Design and Participants

Under the Hong Kong Jockey Club SMART Family-Link Project, the online Family amidst COVID-19 (FamCov) survey was conducted to include as large a sample as possible under budget constraint and rapidly within 6 days during 26–31 May 2020, the easing period after the second COVID-19 wave. Details of the methods have been reported elsewhere [19]. In brief, a well-known survey agency, namely Hong Kong Public Opinion Research Institute (HKPORI), sent email invitations to its probability- and non-probability-based online panels of Hong Kong residents aged 18 years or above to complete an online anonymous self-administrated questionnaire [27]. HKPORI has executed over 1800 independent public opinion surveys since 1991 for organizations including academic institutions and government departments [27]. With no validated scales available on the use of IM functions in family e-chat groups, we designed the questions and conducted pilot tests. No difficulties or sensitive issues were reported by pilot respondents, which supported face validity [19]. Twenty thousand, one hundred and three invitation emails were opened, and 4944 respondents voluntarily completed the self-administered survey (24.6% response rate). After excluding respondents having no family members (*n* = 30) and those having over 30% missing values (*n* = 24), 4890 respondents (98.9%) were included in the present study.

The study was carried out in accordance with the guidelines and regulations laid down in the Declaration of Helsinki. Ethics approval was granted by the Institutional Review Board of the University of Hong Kong/Hospital Authority Hong Kong West Cluster (Reference number: UW 20-238). Participants gave written informed consent before answering the online questionnaire, including the use of the participants’ data for research. Only anonymous data were used in this study.

### 2.2. Measurement

#### 2.2.1. Exposure Measures

Definitions of family (“family members who are related through biological, marital, cohabitation, and/or emotional bonding”), and IM e-chat group (“a group of 3 or more people in IM communication applications, such as WhatsApp or WeChat, etc.”) were provided before the questions. The use of family e-chat groups was asked by the question “Do you have family e-chat groups?” with responses of “Yes” and “No”. Those who selected “Yes” were asked further details when the COVID-19 outbreak was severe: (1) the number of family e-chat groups they had; (2) the IM functions they usually used in family e-chat groups with responses of “Receive/Send text messages”, “Receive/Send photos/pictures”, “Receive/Send short videos”, “Voice messages”, “Video chat”, and “Real-time conversation”; and (3) the average numbers of IM messages received and sent daily in family e-chat groups separately with responses categorized into “<1 message”, “1–2 messages”, “3–10 messages”, “11–20 messages”, and “over 20 messages”. The questions on the numbers of IM messages have been used before [14].

#### 2.2.2. Outcome Measures

The Family Wellbeing Scale was developed and validated in previous studies under the FAMILY project [28,29]. It consists of three separate items of family health, harmony, and happiness (3Hs) using the questions “how healthy/harmonious/happy do you think your family is?”, each measured on an 11-point scale ranging from 0 to 10. A composite score of family wellbeing (range 0–10) was calculated as the total score of family 3Hs divided by 3, with higher scores indicating higher levels of family wellbeing. Family communication quality was assessed using a single item of “How do you find the quality of communication between you and your family members?” on an 11-point scale (0 = very poor, 10 = very good), which has been used in a previous study [30]. Personal happiness was assessed using a single item of “How happy do you think you are?” on an 11-point scale (0 = very unhappy, 10 = very happy), which was found to be reliable and valid in surveys [31].

#### 2.2.3. Covariates

Face-to-face communication was examined by “How many days did you have face-to-face communication with family members on average per week when the COVID-19 outbreak was severe?”, with responses ranging from 0 to 7 days. Information of demographic and socioeconomic characteristics was also collected, including sex, age group (18–24 years, 25–34 years, 45–64 years, and 65 years or above), education (primary or below, secondary, post-secondary, and college degree or above), monthly household income (no income, less than HK4000, HK4000–9999, HK10,000–19,999, HK20,000–29,999, HK30,000–39,999, and HK40,000 or higher) (US1.0 = HK7.8), household size (number of people living together, including the respondent), and housing type (rented and owned).

#### 2.2.4. Statistical Analysis

Monthly household income was dichotomized into lower and higher according to the household size and the median household income from the 2019 census data in Hong Kong [32]. Socioeconomic status was a composite score of education (0 = secondary or below, 1 = tertiary), income (0 = lower, 1 = higher), and housing (0 = rented, 1 = owned), and analyzed as low (0–1), medium (2), and high (3) [19].

Data were weighted by sex, age, and education attainment according to the 2019 Hong Kong census data [33,34]. A Chi-square test was used to compare the characteristics of people with and without family e-chat groups. Cramer’s V indicated the effect size of categorical variables, which was calculated by taking the square root of the chi-squared statistics divided by the sample size and the minimum number of categories of row or column minus 1 [35]. Poisson regression models with robust variance estimators yielded adjusted prevalence ratios (aPRs) and 95% confidence intervals (CIs) for different IM functions used in family e-chat groups in relation to sex, age group, socioeconomic status, and number of days having face-to-face communication with family/week [36], with mutual adjustments of different functions, since some respondents used several functions. Regression coefficient (β) and 95% CIs were calculated using multivariable linear regressions to examine the associations of number of family e-chat groups (all respondents) and use of IM functions (no family e-chat groups excluded) with family communication, family wellbeing, and personal happiness, adjusted for sex, age, socioeconomic status, and number of days having face-to-face communication with family/week. The Sobel–Goodman mediation test was used to examine the mediating (indirect) effect of family communication in the associations of number of family e-chat groups used (all respondents) and IM functions used (no family e-chat groups excluded) with family wellbeing and personal happiness. Bias-corrected bootstrapping with 1000 replications was used to calculate the 95% CIs of indirect and direct effects, adjusted for sex, age, socioeconomic status, and number of days having face-to-face communication with family/week. All analyses were conducted using STATA version 15.0 (StataCorp LP, College Station, TX, USA). A 2-sided *p* < 0.05 was considered statistically significant.

## 3. Results

Table 1 shows that, after weighting, 52.9% of respondents were female, 37.7% were aged 45–64 years, and 21.3% ≥65 years, 65.7% had secondary or below education, 52.6% had lower monthly household income, 63.4% lived in owned housing, and 33.3% and 14.4% had medium and high socioeconomic status, respectively. For respondents having family e-chat groups, 55.9% were female, 38.5% were aged 45–64 years, and 22.1% ≥65 years, 66.1% had secondary or below education, 52.1% had lower monthly household income, 65.4% lived in owned housing, and 33.8% and 14.5% had medium and high socioeconomic status, respectively.

Table 2 shows that, after weighting, 16.1% of respondents had no family e-chat groups, and 34.4% had three or more groups. The most common function used in family e-chat groups was receiving/sending text messages (78.4%), followed by receiving/sending photos/pictures (76.5%), making voice calls (46.2%), receiving/sending short videos (37.2%), and voice messages (13.8%), and making video calls (8.2%). Forty-eight point three percent of respondents used three or more functions in family e-chat groups, and 93.0% and 89.6% received and sent at least one IM daily, respectively.

Table 3 shows that more female and older respondents used three or more IM functions in family e-chat groups (aPRs 1.04 to 1.22, all *p* ≤ 0.001). More women reported making voice calls (aPR 1.11, 95% CI 1.02–1.21, *p* = 0.02), receiving/sending short videos (aPR 1.10, 95% CI 1.00–1.20, *p* = 0.047), receiving/sending voice messages (aPR 1.27, 95% CI 1.07–1.52, *p* = 0.007), and making video calls (aPR 1.31, 95% CI 1.05–1.65, *p* = 0.02). Older respondents, especially those aged ≥65 years, used more non-text functions, including receiving/sending photos/pictures, voice calls, receiving/sending short videos, and video calls (aPRs 1.17 to 3.10, all *p* for trend ≤0.03). Fewer women and respondents aged ≥65 years reported receiving/sending text messages (aPRs 0.97 and 0.86, respectively, both *p* ≤ 0.02). More respondents having higher socioeconomic status received/sent text messages (aPRs: medium 1.06 and high 1.08) and fewer received/sent voice messages (aPRs: medium 0.78 and high 0.72) (both *p* for trend ≤0.004).

Table 4 shows that receiving/sending photos/pictures (adjusted β = 0.39), making voice calls (adjusted β = 0.25), receiving/sending voice messages (adjusted β = 0.23), and making video calls (adjusted β = 0.50) were associated with better family communication (all *p* ≤ 0.03). Receiving/sending photos/pictures, making voice calls, and making video calls were associated with higher levels of family wellbeing (adjusted βs 0.18 to 0.45, all *p* ≤ 0.003). Only making voice calls and video calls were associated with higher scores of personal happiness (adjusted βs 0.30 and 0.32, respectively, both *p* ≤ 0.009).

Table 5 shows that having more family e-chat groups, using more IM functions, and receiving and sending more IM messages daily in family e-chat groups were associated with better family communication (adjusted βs 0.32 to 0.83, all *p* ≤ 0.01) and family wellbeing (adjusted βs 0.27 to 0.77, all *p* ≤ 0.004), and higher personal happiness (adjusted βs 0.30 to 0.72, all *p* ≤ 0.03) (all *p* for trend ≤0.02).

Family communication partially mediated the associations of having more family e-chat groups, and more IM functions used in family e-chat groups with family wellbeing (proportion of total effects mediated: 75.5% and 66.4%, respectively) and personal happiness (proportion mediated: 69.5% and 46.2%, respectively) (Sobel–Goodman test: *p* < 0.001) (Table 6).

## 4. Discussion

This is the first report showing that receiving/sending photos/pictures and making video calls were independently associated with family communication quality, family wellbeing, and personal happiness. We also first reported that having more family e-chat groups and using more IM functions in family e-chat groups had dose–response associations with higher levels of family wellbeing and higher personal happiness. About half to three-quarters of these associations were mediated by family communication quality.

The mediating effects of family communication quality add new evidence to the theoretical frameworks [16,24]. Previous studies mainly focused on the effects of social networking sites and social media use on personal life satisfaction and psychological health in young people [37,38,39]. We showed that family e-chat groups used in closed communication circles were associated with better wellbeing when face-to-face communication was restrained during the pandemic. Using IM functions may protect families and individuals from the risks and mental burdens of the pandemic through better communication quality.

Both photo/picture messaging and video calls are methods of visual interpersonal communication. Enriched communication channels can facilitate social interaction to create closer interpersonal social relationships [40]. Emoji and pictures in IM are widely popular, which incorporate playful elements into a plain message to attract receivers’ attention, vividly express personal emotion, and thus facilitate communication effect [40,41]. Photo messaging enables users to timely share memorable moments in daily life with all family members, especially those being geographically separated or across generations, which has been shown to enhance intimate family communication [42].

Despite the overlapping 95% CIs with other IM functions, video calls appeared to be most strongly associated with higher levels of family wellbeing. This is consistent with a previous study in 2016, which showed sharing family life information through video calls was associated with much higher levels of family wellbeing [13]. The present study further reported its strong associations with better family communication and personal happiness. Amidst the COVID-19 pandemic, almost all face-to-face social activities are regarded as high risk. Family members of all generations faced elevated social isolation due to the physical distancing and lockdown policy. Instead of one-on-one in-person communications, online group settings allow more effective and simultaneous information exchange and interactions among many separated family members, which can evoke warm feelings of family gathering and close connection when face-to-face gatherings are impossible. Family video calls can partly overcome the barrier to traditional family reunions, such as birthday parties or other celebrations [16]. Even the inactive family members and those who live far away can participate in and enjoy the online gathering time. The physical distancing due to COVID-19 could have motivated more people to use video calls to reduce emotional distancing within the family.

Although women and older people had less access to smartphones and the Internet in Hong Kong [26], they had more family e-chat groups and used more non-text IM functions amidst the COVID-19 pandemic. A web-based survey in the Netherlands found more men than women used the Internet for COVID-19–related communications, but it was not restricted to the use of family e-chat groups [43]. In line with our results, several studies have reported that women used more digital communication to interact with family before or amidst the pandemic, especially video calls [13,14,44,45]. Such behaviors could be explained by perceived usefulness and enjoyment, attachment motivation, and relationship commitment, which were associated with the adoption of IM communication [46]. The COVID-19 pandemic has increased the care burden for women [47], and Chinese women were found to perceive a higher level of family demands than men [48]. Non-text functions through photos, voice, and video interaction could partially make up for the lack of face-to-face communication, provide greater communication satisfaction over distance [44], and thus preferred by women to help maintain their roles in care activities and connectedness with remote family members. In addition, older people show more care for family affairs and view family communication as being worthy of time and dedication [49]. Elderly family members, such as grandparents, are believed to have more barriers in accessing digital functions [50]. However, they attach greater importance to digital communication and use smartphones increasingly [26,51]. To fit into younger family members’ schedules, they have shown a willingness to adopt new communication media [52]. Compared with text messaging, non-text functions in family e-chat groups are more receptive as being easier to use, especially for older people who may have difficulties in text typing [52].

We also found more family e-chat groups and IM function use being associated with higher personal happiness and the mediating effects of family communication quality. Previous studies have reported that IM use had no associations with emotionally closer relationships and happiness [53,54], while the present study stressed the importance of family communication and provided the first evidence linking more IM use in family e-chat groups with happiness amidst the COVID-19 pandemic. Family members have interconnections and influence each other’s functioning [55]. According to the attachment theory, pleasant and frequent interactions with others are critical for personal mental and emotional wellbeing [46]. Better family communication can provide support for individuals to manage stress [16,56] and maintain personal wellbeing [57]. Our finding also offered support to the media naturalness hypothesis, which posits effective communication modes with visual or vocal cues improve positive interaction and relationships [54,58].

Family wellbeing and personal happiness have a mutually reinforcing linkage, whereby the achievement and disruptiveness in one begets the same in the other [16]. Family wellbeing is valued above personal happiness across cultures and is the foundation to individual family members’ happiness across the lifespan [1]. Meanwhile, the level of personal happiness may differ across family members and could inversely affect one’s perception of family wellbeing [59]. Quality family communication is crucial for both.

While we have discussed the positive aspects of the above associations, our results also suggest that those without or with low use of family e-chat groups could be vulnerable. Policymakers and social health care professionals need to pay special attention to these risk factors and provide interventions and assistance amidst the pandemic.

Our study had some limitations. First, recall errors were an inevitable but random error of self-reported family e-chat group use would have led to under-estimated effect size. Second, better family relationships and higher communication quality could also promote more use of family e-chat groups to keep connected. Reverse causality was possible due to the cross-sectional survey design. However, to provide a clearer temporal sequence, we asked the respondents to report their ICTs use when the COVID-19 outbreak was severe and their perceived family communication quality, wellbeing, and personal happiness during the easing period. Prospective studies are needed to confirm such associations. Third, considering the dynamic and unpredictable changes of the COVID-19 pandemic, we tried to collect the largest sample possible within a short period and a constrained budget. The included respondents were younger and better educated than the general population in Hong Kong. The prevalence, even after weighting, might not be generalizable to the general population. However, because only small differences were found between the unweighted and weighted prevalence of use of family e-chat groups, selection bias would not have substantial influences on the observed associations. We also reported the dose–response associations between IM messages received/sent in family e-chat groups per day and family wellbeing, being consistent with previous findings [14], which would support our results. Fourth, although family members may tend to share family, health, and epidemic-related information in family e-chat groups, we did not ask about the delivered or shared contents as the questionnaire was already quite long. Future studies are warranted. Lastly, although we showed the benefits of using e-chat groups for family communication amidst the pandemic, heavy use of digital platforms, including social media, may reduce the opportunities of face-to-face communication and lead to loneliness, reduced social connectedness, and other psychosocial problems [60]. Family e-chat groups should be used to complement face-to-face communication, not to replace it.

## 5. Conclusions

We have first reported that amidst the COVID-19 pandemic, having more family e-chat groups, using more IM functions, such as sending/receiving photos/pictures and making video calls in family e-chat groups, were associated with higher levels of family wellbeing and personal happiness, and about half to three-quarters of the associations were mediated by family communication quality. Prospective studies are needed to confirm the associations. People without or with low use of family e-chat groups amidst the pandemic would need more attention and assistance in the presence of social distancing.

## Figures and Tables

**Table 1:** Prevalence of having family e-chat groups by sociodemographic characteristics of respondents, *n* (%).

Demographics	Total, *n* (%) (*n* = 4890)		Effect Size ^c^	Having Family E-Chat Groups, *n* (%) (*n* = 4046)		Effect Size ^c^
	Unweighted ^a^	Weighted ^b^		Unweighted ^a^	Weighted ^b^	
Sex			0.03			0.02
Male	2138 (43.7)	2295 (47.1)		1721 (42.5)	1806 (44.2)	
Female	2752 (56.3)	2583 (52.9)		2325 (57.5)	2285 (55.9)	
Age group, years			0.29			0.29
18–24	219 (4.5)	416 (8.5)		158 (3.9)	302 (7.4)	
25–44	2449 (50.1)	1581 (32.4)		1990 (49.2)	1307 (32.0)	
45–64	2013 (41.2)	1839 (37.7)		1714 (42.4)	1577 (38.5)	
≥65	210 (4.3)	1041 (21.3)		184 (4.6)	905 (22.1)	
Education			0.53			0.47
Secondary/below	659 (13.6)	3183 (65.7)		561 (14.0)	2688 (66.1)	
Tertiary	4199 (86.4)	1662 (34.3)		3457 (86.0)	1376 (33.9)	
Monthly household income		0.23			0.24	
Lower	1270 (29.8)	2201 (52.6)		1014 (28.7)	1832 (52.1)	
Higher	2986 (70.2)	1986 (47.4)		2524 (71.3)	1685 (47.9)	
Housing type			0.14			0.02
Rented	1603 (33.9)	1744 (36.6)		1265 (32.3)	1388 (34.6)	
Owned	3120 (66.1)	3025 (63.4)		2653 (67.7)	2628 (65.4)	
Socioeconomic status ^d^		0.40			0.41	
Low	790 (18.9)	2160 (52.3)		636 (18.3)	1802 (51.7)	
Medium	1497 (35.8)	1375 (33.3)		1215 (34.9)	1177 (33.8)	
High	1891 (45.3)	595 (14.4)		1632 (46.9)	505 (14.5)	

^a^ Respondents with missing data were excluded. ^b^ Weighted by sex, age, and education of the 2019 Hong Kong census data. ^c^ Cramer’s V: 0.10–0.30, small; 0.30–0.50, medium; ≥0.50, large. ^d^ Socioeconomic status: a composite score of education (0 = secondary or below, 1 = tertiary), income (0 = lower, 1 = higher), and housing (0 = rented, 1 = owned), analyzed as low (0–1), medium (2) and high (3).

**Table 2:** Number of family e-chat groups (*n* = 4890) and use of IM functions (*n* = 4046) ^a^ when COVID-19 outbreak was severe, *n* (%).

Use of Family E-Chat Groups	Unweighted Prevalence ^b^	Weighted Prevalence ^c^	Effect Size ^d^
Number of family e-chat groups			0.02
0	844 (17.3)	786 (16.1)	
1	1162 (23.8)	1112 (22.8)	
2	1287 (26.3)	1301 (26.7)	
≥3	1597 (32.7)	1678 (34.4)	
Receiving/sending text messages			0.02
Yes	3224 (82.3)	3110 (78.4)	
No	693 (17.7)	855 (21.6)	
Receiving/sending photos/pictures			<0.001
Yes	3054 (78.0)	3032 (76.5)	
No	863 (22.0)	934 (23.5)	
Making voice calls			0.08
Yes	1520 (38.8)	1833 (46.2)	
No	2397 (61.2)	2133 (53.8)	
Receiving/sending short videos			0.02
Yes	1391 (35.5)	1474 (37.2)	
No	2526 (64.5)	2491 (62.8)	
Receiving/sending voice messages			0.001
Yes	541 (13.8)	549 (13.8)	
No	3376 (86.2)	3417 (86.2)	
Making video calls			0.008
Yes	346 (8.8)	324 (8.2)	
No	3571 (91.2)	3641 (91.8)	
Number of IM functions used			0.03
0–1	908 (22.5)	933 (22.8)	
2	1255 (31.0)	1178 (28.8)	
≥3	1880 (46.5)	1975 (48.3)	
Number of IM messages received daily			0.05
<1	273 (7.0)	275 (7.0)	
1–2	963 (24.8)	1007 (25.6)	
3–10	1826 (47.0)	1818 (46.2)	
11–20	481 (12.4)	500 (12.7)	
>20	345 (8.9)	339 (8.6)	
Number of IM messages sent daily			0.04
<1	424 (11.3)	398 (10.4)	
1–2	1313 (35.1)	1331 (34.7)	
3–10	1552 (41.5)	1611 (42.0)	
11–20	276 (7.4)	259 (6.8)	
>20	174 (4.7)	239 (6.2)	

^a^ IM: instant messaging. Respondents having no family e-chat groups (*n* = 844) were excluded. ^b^ Respondents with missing data were excluded. ^c^ Weighted by sex, age, and education of the 2019 Hong Kong census data. ^d^ Cramer’s V: 0.10–0.30, small; 0.30–0.50, medium; ≥0.50, large.

**Table 3:** Associations of sociodemographic characteristics with the number of IM functions and their use in family e-chat groups (*n* = 4046), aPR (95% CI) ^a^.

Characteristics	Using ≥3 IM Functions ^b^	Receiving/Sending Text Messages	Receiving/Sending Photos/Pictures	Making Voice Calls	Receiving/Sending Short Videos	Receiving/Sending Voice Messages	Making Video Calls
Sex							
Male	1	1	1	1	1	1	1
Female	1.04 (1.01, 1.06) ^e^	0.97 (0.94, 1.00) ^d^	1.04 (1.00, 1.07)	1.11 (1.02, 1.21) ^d^	1.10 (1.00, 1.20) ^d^	1.27 (1.07, 1.52) ^e^	1.31 (1.05, 1.65) ^d^
Age group (year)							
18–24	1	1	1	1	1	1	1
25–44	1.07 (1.00, 1.14)	0.97 (0.90, 1.05)	1.11 (0.98, 1.26)	1.17 (0.87, 1.57)	1.62 (1.07, 2.46) ^d^	1.31 (0.79, 2.16)	2.22 (0.93, 5.32)
45–64	1.18 (1.10, 1.26) ^f^	0.93 (0.86, 1.00)	1.17 (1.03, 1.33) ^d^	1.50 (1.12, 2.01) ^e^	2.71 (1.79, 4.11) ^f^	1.11 (0.67, 1.85)	1.83 (0.76, 4.39)
65+	1.22 (1.12, 1.32) ^f^	0.86 (0.77, 0.97) ^d^	1.19 (1.03, 1.37) ^d^	2.24 (1.64, 3.05) ^f^	2.90 (1.86, 4.51) ^f^	1.28 (0.69, 2.38)	3.10 (1.20, 8.00) ^d^
*p* for trend	<0.001	0.007	0.01	<0.001	<0.001	0.53	0.03
Socioeconomic status ^c^							
Low	1	1	1	1	1	1	1
Medium	1 (0.97, 1.03)	1.06 (1.01, 1.12) ^d^	1.01 (0.96, 1.07)	0.90 (0.80, 1.01)	1.03 (0.91, 1.18)	0.78 (0.62, 0.98) ^d^	0.90 (0.66, 1.23)
High	1 (0.97, 1.04)	1.08 (1.03, 1.13) ^e^	1.01 (0.96, 1.06)	0.90 (0.81, 1.01)	1.05 (0.93, 1.19)	0.72 (0.58, 0.90) ^e^	0.89 (0.66, 1.20)
*p* for trend	0.82	0.002	0.73	0.07	0.44	0.004	0.45

^a^ Respondents having no family e-chat groups (*n* = 844) were excluded. aPR (95% CI): adjusted prevalence ratio (95% confidence intervals), sex, age group, and socioeconomic status were mutually adjusted. ^b^ IM: instant messaging. ^c^ Socioeconomic status: a composite score of education (0 = secondary or below, 1 = tertiary), income (0 = lower, 1 = higher), and housing (0 = rented, 1 = owned), analyzed as low (0–1), medium (2) and high (3). ^d^ *p* < 0.05; ^e^ *p* < 0.01; ^f^ *p* < 0.001.

**Table 4:** Associations of IM functions used in family e-chat groups with family communication, family wellbeing, and personal happiness (*n* = 4046) ^a^.

IM Functions Used in Family E-Chat Groups ^a^	Family Communication ^b^		Family Wellbeing ^b^		Personal Happiness ^b^	
	Mean ± SD ^c^	Adjusted β (95% CI) ^d^	Mean ± SD ^c^	Adjusted β (95% CI) ^d^	Mean ± SD ^c^	Adjusted β (95% CI) ^d^
Receiving/sending text messages						
No	6.6 ± 2.0	0	7.1 ± 1.6	0	6.0 ± 2.1	0
Yes	6.6 ± 1.9	0.04 (−0.14, 0.23)	7.2 ± 1.6	0.03 (−0.13, 0.19)	6.2 ± 2.0	0.11 (−0.10, 0.32)
Receiving/sending photos/pictures						
No	6.2 ± 2.1	0	6.9 ± 1.7	0	5.8 ± 2.2	0
Yes	6.7 ± 1.8	0.39 (0.22, 0.55) ^g^	7.3 ± 1.5	0.33 (0.20, 0.47) ^g^	6.2 ± 2.0	0.30 (0.12, 0.49) ^f^
Making voice calls						
No	6.5 ± 1.9	0	7.1 ± 1.6	0	6.1 ± 2.0	0
Yes	6.9 ± 1.8	0.25 (0.11, 0.39) ^g^	7.7 ± 1.3	0.18 (0.06, 0.30) ^f^	6.2 ± 2.1	0.03 (−0.13, 0.18)
Receiving/sending short videos						
No	6.5 ± 1.9	0	7.1 ± 1.6	0	6.0 ± 2.0	0
Yes	6.9 ± 1.7	0.04 (−0.10, 0.18)	7.3 ± 1.5	−0.06 (−0.18, 0.05)	6.3 ± 2.0	−0.12 (−0.27, 0.04)
Receiving/sending voice messages						
No	6.6 ± 1.9	0	7.1 ± 1.6	0	6.1 ± 2.0	0
Yes	7.0 ± 1.8	0.23 (0.05, 0.41) ^e^	7.4 ± 1.5	0.11 (−0.05, 0.26)	6.2 ± 2.1	−0.01 (−0.21, 0.19)
Making video calls						
No	6.6 ± 1.9	0	7.1 ± 1.6	0	6.1 ± 2.0	0
Yes	7.3 ± 1.5	0.50 (0.28, 0.72) ^g^	7.7 ± 1.3	0.45 (0.27, 0.64) ^g^	6.5 ± 2.0	0.32 (0.08, 0.57) ^f^

^a^ IM: instant messaging. Respondents having no family e-chat groups (*n* = 844) were excluded. ^b^ Range 0–10, higher scores indicate better outcomes. ^c^ SD: standard deviation. ^d^ CI: confidence intervals. Adjusted for sex, age, socioeconomic status, number of days having face-to-face communication with family/week, and mutually adjusted for each other. ^e^ *p* < 0.05; ^f^ *p* < 0.01, ^g^ *p* < 0.001.

**Table 5:** Associations of the number of family e-chat groups (*n* = 4890) and the use of IM functions (*n* = 4046) with family communication, family wellbeing, and personal happiness.

Use of Family E-Chat Groups	Family Communication ^b^		Family Wellbeing ^b^		Personal Happiness ^b^	
	Mean ± SD ^c^	Adjusted β (95% CI) ^d^	Mean ± SD ^c^	Adjusted β (95% CI) ^d^	Mean ± SD ^c^	Adjusted β (95% CI) ^d^
Number of family e-chat groups		<0.001		<0.001		<0.001
0		0		0		0
1	6.1 ± 2.2	0.15 (−0.03, 0.34)	6.7 ± 1.8	0.23 (0.07, 0.38) ^f^	5.7 ± 2.2	0.30 (0.10, 0.50) ^f^
2	6.6 ± 1.9	0.52 (0.34, 0.70) ^g^	7.1 ± 1.6	0.51 (0.35, 0.66) ^g^	6.1 ± 2.0	0.56 (0.37, 0.76) ^g^
≥3	6.9 ± 1.7	0.83 (0.65, 1.00) ^g^	7.4 ± 1.4	0.77 (0.63, 0.92) ^g^	6.3 ± 1.9	0.72 (0.53, 0.90) ^g^
*p* for trend		<0.001		<0.001		<0.001
Number of IM functions used ^a^		<0.001		<0.001		<0.001
≤1	6.0 ± 2.3	0	6.7 ± 1.8	0	5.7 ± 2.2	0
2	6.5 ± 1.8	0.48 (0.31, 0.64) ^g^	7.1 ± 1.5	0.43 (0.29, 0.57) ^g^	6.1 ± 2.0	0.42 (0.24, 0.60) ^g^
3	6.9 ± 1.7	0.72 (0.56, 0.88) ^g^	7.3 ± 1.5	0.51 (0.38, 0.64) ^g^	6.3 ± 2.0	0.36 (0.18, 0.53) ^g^
*p* for trend		<0.001		<0.001		<0.001
Number of IM messages received daily ^a^		<0.001		<0.001		0.01
<1	6.1 ± 2.2	0	6.8 ± 1.8	0	5.9 ± 2.1	0
1–2	6.4 ± 2.0	0.15 (−0.12, 0.42)	7.0 ± 1.6	0.09 (−0.14, 0.31)	6.0 ± 2.0	0.05 (−0.25, 0.34)
3–10	6.7 ± 1.8	0.32 (0.07, 0.57) ^e^	7.2 ± 1.5	0.21 (0, 0.43)	6.2 ± 2.0	0.14 (−0.14, 0.42)
11–20	7.0 ± 1.7	0.61 (0.32, 0.90) ^g^	7.5 ± 1.5	0.46 (0.21, 0.71) ^g^	6.4 ± 2.0	0.42 (0.10, 0.74) ^e^
>20	7.0 ± 1.8	0.64 (0.33, 0.95) ^g^	7.4 ± 1.5	0.38 (0.11, 0.64) ^f^	6.1 ± 2.1	0.25 (−0.09, 0.60)
*p* for trend		<0.001		<0.001		0.02
Number of IM messages sent daily ^a^		<0.001		<0.001		<0.001
<1	6.3 ± 2.0	0	6.9 ± 1.7	0	6.0 ± 2.1	0
1–2	6.5 ± 1.9	0.16 (−0.05, 0.37)	7.1 ± 1.6	0.13 (−0.05, 0.30)	6.0 ± 2.0	0 (−0.23, 0.24)
3–10	6.8 ± 1.8	0.45 (0.25, 0.66) ^g^	7.3 ± 1.5	0.27 (0.09, 0.44) ^f^	6.2 ± 2.0	0.13 (−0.10, 0.35)
11–20	7.2 ± 1.7	0.82 (0.54, 1.11) ^g^	7.5 ± 1.5	0.54 (0.30, 0.79) ^g^	6.6 ± 2.0	0.67 (0.35, 0.99) ^g^
>20	7.2 ± 1.7	0.79 (0.46, 1.12) ^g^	7.5 ± 1.5	0.48 (0.20, 0.76) ^f^	6.1 ± 2.1	0.14 (−0.23, 0.51)
*p* for trend		<0.001		<0.001		0.02

^a^ IM: instant messaging. Respondents having no family e-chat groups (*n* = 844) were excluded. ^b^ Range 0–10, higher scores indicate better outcomes. ^c^ SD: standard deviation. ^d^ CI: confidence intervals. Adjusted for sex, age, socioeconomic status, and number of days having face-to-face communication with family/week. ^e^ *p* < 0.05; ^f^ *p* < 0.01; ^g^ *p* < 0.001.

**Table 6:** Adjusted indirect, direct, and total effect of number (*n* = 4890) of family e-chat groups and IM functions used (*n* = 4046) ^a^ on family wellbeing and personal happiness mediated by family communication quality.

	Family Wellbeing ^b^				Personal Happiness ^b^			
	Adjusted β ^c^	Boot SE ^d^	*p*	Boot 95% CI ^e^	Adjusted β ^c^	Boot SE ^d^	*p*	Boot 95% CI ^e^
Number of family e-chat groups								
Total effect	0.26	0.024	<0.001	0.21, 0.31	0.23	0.029	<0.001	0.18, 0.29
Indirect effect (via mediation)	0.20	0.019	<0.001	0.16, 0.24	0.16	0.016	<0.001	0.13, 0.19
Direct effect (without mediation)	0.06	0.014	<0.001	0.04, 0.09	0.07	0.025	<0.001	0.02, 0.12
Proportion of total effect mediated	75.5%				69.5%			
Number of IM functions used ^a^								
Total effect	0.32	0.077	<0.001	0.17, 0.47	0.38	0.102	<0.001	0.18, 0.58
Indirect effect (via mediation)	0.21	0.063	<0.001	0.09, 0.34	0.18	0.056	<0.001	0.07, 0.29
Direct effect (without mediation)	0.11	0.046	0.006	0.02, 0.20	0.21	0.093	0.02	0.02, 0.37
Proportion of total effect mediated	66.4%				46.2%			

^a^ IM: instant messaging. Respondents having no family e-chat groups (*n* = 844) were excluded. ^b^ Range 0–10, higher scores indicate better outcomes. ^c^ Adjusted for sex, age, socioeconomic status, and number of days having face-to-face communication with family/week. ^d^ SE: Bias-corrected standard error, calculated using bootstrap methods with 1000 replications. ^e^ CI: Bias-corrected confidence intervals, calculated using bootstrap methods with 1000 replications.

## Data Availability

The data presented in this study are available on request from the corresponding authors. The data are not publicly available because our analyses and paper writing on the results are in progress.
